# Pharmaceutically Assisted Angioplasty to Relieve Cerebral Vasospasm after Aneurysmal Subarachnoid Hemorrhage—A Retrospective, Single-Center, Observational Comparison of Established and New Treatment Techniques

**DOI:** 10.1007/s00270-026-04367-w

**Published:** 2026-02-16

**Authors:** C. Thole, C. J. Maurer, A. Berlis

**Affiliations:** 1 Department of Diagnostic and Interventional Neuroradiology, University Hospital Augsburg, Augsburg, Germany; 2 Department of Radiology, Neuroradiology and Interventional Radiology, BG Trauma Center Murnau, Murnau, Germany

## Abstract

**Purpose:**

To compare differences in efficacy of endovascular vasospasm therapy (EVT) with pharmacological, mechanical or combined treatment.

**Methods:**

In a single-center retrospective analysis, we included 60 patients with EVT after aneurysmal subarachnoid hemorrhage from 2017–2022. EVT treatment groups were: 1. Single or 2. dual vasodilator application, 3. transluminal balloon angioplasty (TBA) alone, with 4. single, or 5. dual vasodilator application, or 6. Stent-Retriever-Angioplasty alone, with 7. single, or 8. dual vasodilator application. Arterial diameters were measured on admission and after treatment. We compared relative increase and significant difference in diameter for each treatment group via multivariable linear regression (SPSS statistics). Age, sex, number of previous treatments and severity of spasm were included as confounding variables.

**Results:**

In 628 treated vessel segments, dual vasodilator application showed significant difference restoring 4.4% more diameter than single vasodilator application alone (*p*—.015). TBA alone, with single or dual vasodilator application was most effective, restoring 28.4% more diameter than single vasodilator application alone (*p* < .001). Stent-Retriever-Angioplasty was more effective with combined dual vasodilator application (*p* < .001), not with single or no vasodilator combination. Adverse outcomes were recorded in 2.4% of interventions.

**Conclusion:**

EVT effectiveness remains a key target in vasospasm rescue treatment but relies on the right treatment modality. TBA remains most effective in vessel dilation. Stent-Retriever-Angioplasty may present an alternative to just pharmacological treatment balancing accessibility and risk profile. Using a combination of vasodilators may enhance treatment efficacy but the combination of nimodipine and papaverine shows little effect in our study.

**Graphical Abstract:**

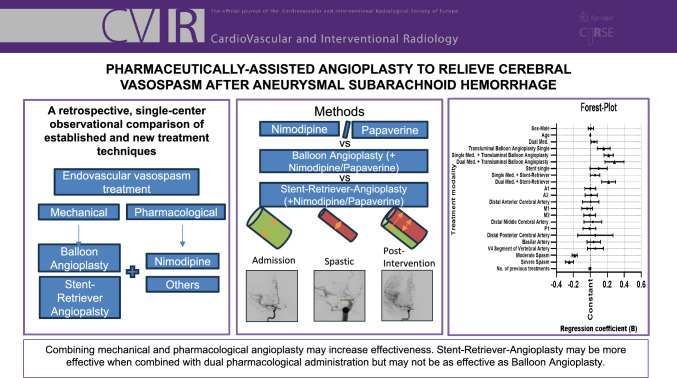

**Supplementary Information:**

The online version contains supplementary material available at 10.1007/s00270-026-04367-w.

## Introduction

Vasospasm is typically defined as a reduction in the diameter of cerebral blood vessels with possible neurologic deterioration and cerebral ischemia (DCI) [1]. After aneurysmal subarachnoid hemorrhage (aSAH), vasospasms manifest between 7 and 10, but up to 21 days after aSAH, and are usually screened for via transcranial Doppler sonography (TCD), CT perfusion imaging or MRI. [2, 3] Therapeutic options include the use of pharmacological agents such as intra-arterial nimodipine or papaverine, endovascular angioplasty with transluminal balloon angioplasty (TBA) or, more recently, vessel dilation with stent-retriever angioplasty. Untreated, vasospasm can lead to DCI. The exact pathogenesis of DCI remains unclear with cortical depolarization, inflammation and pro-vasoconstrictive mediators being investigated for their effect. Large-vessel vasospasm is no longer suspected to be the only predictor of DCI and unfavorable outcome as small vessel spasm, ICU treatment and inflammatory cascade seem influential, but vasospasm remains a therapeutic target in prophylaxis of DCI. [2, 4].

Despite the efficacy of vasospasm treatment in improving patient outcomes, the considerable heterogeneity in management approaches has resulted in a dearth of evidence-based therapeutic regimens to relieve vasospasm. Orally administered nimodipine remains the only evidence-based prophylactic agent with recommendation in the current guidelines [1]. Endovascular treatment options may improve outcome of patients due to the potential for more effective effect of vessel dilation [5, 6]. The introduction of stent-retriever angioplasty has expanded treatment options, but there is currently a lack of direct comparisons between different endovascular vasospasm treatment (EVT) techniques. Stent-retrievers work by exerting less radial force than TBA-Balloons when released and as such may cause less damage in smaller vessel compared to TBA. [6] While previous studies have examined the effects of pharmacological or stent-retriever angioplasty, there are no large-scale studies that compare these treatment methods when used in combination or assess their impact on angiographic outcomes [6–9]. Therapy-resistant, angiographic vasospasm is associated with a worse patient outcome and as such presents a target for novel developing treatment techniques [10].

The objective of this study is to investigate the technical differences in angiographic efficacy with the use of different treatment techniques and their combinations. We present a large collection of angiographic documentation of treated vessel segments, allowing for a comprehensive comparison of combinations of pharmacological and mechanical techniques.

## Methods

In this single-center, retrospective cohort study, we collected data of all patients who received interventional EVT after aSAH in a single medical center in (–) during the inclusion period between January 2017 and December 2022. Approval from the local ethics committee (Ethics committee of the -/-university of -) was obtained.

Inclusion criteria were EVT interventions resulting from aSAH, availability of digital subtraction angiography (DSA) runs during aneurysm treatment as well as vasospasm treatment to assess the diameter of the treated vessel segments. Eligibility for EVT is defined below. Exclusion criteria were a lack of sufficient angiographic data to assess for vessel diameter and/or EVT for non-aneurysmal SAH.

### Intervention and Treatment Allocation

After aneurysm treatment, oral nimodipine prophylaxis was routinely initiated on day two after aSAH, while the clinical condition was monitored in intensive care unit (ICU) setting. Clinical eligibility for EVT was based on otherwise unexplained neurological deterioration together with TCD evidence of vasospasm, defined as mean flow velocities > 180 cm/s in the anterior circulation or a > 50% increase within 24 h from baseline. Patients received vasospasm treatment no longer than 6 h after diagnosing. During DSA imaging the anterior and posterior circulation of both hemispheres were screened for detection of any spastic vessel segments. The spastic vessel segments were defined as the vessel segments most affected by vasospasm with reduced contrast enhancement of the peripheral circulation as per qualitative determination on angiographic imaging during and after procedure by a board-certified neuroradiologist.

Pharmacological treatment modalities of spastic vessels included single or dual pharmacological therapy with either intra-arterial calcium antagonist nimodipine and/or alkaloid papaverine, or a combination of both. Use of nimodipine can result in intracranial pressure (ICP) fluctuations resulting in papaverine as the first alternative. Intra-arterial dosing was titrated to vasospasm severity with a maximum of 3 mg nimodipine and/or 200 mg papaverine per vascular territory (right/left anterior circulation or vertebrobasilar system).

Treatment allocation—as defined below—was operator-dependent without a formalized algorithm. TBA was generally reserved for vasospasm in the basilar artery, distal internal carotid artery (ICA), and the M1 segment of the middle cerebral artery (MCA) whenever feasible. Stent-retriever angioplasty was furthermore utilized in A2, A3 (Distal anterior cerebral artery (ACA)) M2 and P1 segments whenever feasible. Mechanical angioplasty was generally reserved for moderate to severe spasm (> 50% diameter reduction) but was ultimately operator-dependent. Heparin was routinely administered during TBA because prolonged balloon inflation and vessel manipulation can carry a higher thromboembolic risk, whereas stent-retriever maneuvers were performed without systemic anticoagulation, consistent with established practice in acute stroke procedures where anticoagulation is routinely avoided to reduce hemorrhagic risk. Spastic vessel segments with no peripheral flow reduction that were not consecutively treated were documented but not included in the analysis.

All patients with EVT were subject to strict angiographic monitoring until the condition has been resolved. Patients received control-angiography 24 h after EVT treatment if multiple vessel were treated before. As such, all treatments were recorded with the number of previous treatments also being documented. No follow-up was conducted.

### Data Management

Baseline characteristics included demographic data (age, sex), admission scoring using the Hunt&Hess Neurological Scale, aneurysm location, size and the incidence of additional, non-ruptured aneurysms (available in supplemental).

Based on treatment received, patients were allocated to the following groups: 1. pharmacological treatment with a single vasodilator (Single Med.); 2. pharmacological treatment with dual vasodilator application (Dual Med.); 3. TBA without the use of pharmacological spasmolysis (TBA alone); 4. TBA with single vasodilator application (TBA single); 5. TBA with dual vasodilator application (TBA dual); 6. Stent-retriever angioplasty without the use of pharmacological spasmolysis (Stent alone); 7. Stent-retriever angioplasty with single vasodilator application (Stent single); and 8. Stent-retriever angioplasty with dual vasodilator application (Stent dual).

Further data collected during vasospasm treatment included the number of treatment sessions per patient, the number of spastic and/or treated vessel segments, differentiation between anterior/posterior and right/left hemispheres, and detailed documentation of the spastic vessels.

### Measuring Methods

We documented vessel diameters on three occasions during our study. First on aSAH admission day, where patients routinely receive an angiography of the anterior and posterior circulation. Second, during EVT interventions, where spastic vessel segments were documented and third, after treatment of documented spastic vessel segments.

The diameter of spastic vessel segments was measured at the site most severely affected. The percentage of reduction in proportion to the documented diameter on admission day was calculated to determine severity of vasospasm. Treatment modality was documented, with the exact amount of intra-arterial medication (papaverine or nimodipine) for the specific segment as well as the use of balloon angioplasty or stent-retrieving systems. Treatment efficacy was determined by measuring the restored segments diameter, calculating the percentage of original diameter restored (non-spastic diameter—restored vessel diameter reduction) with the percentage of restoration as a surrogate parameter for improved flow to peripheral vessel territories.

Vasospasm severity was graded categorically, grading diameter reduction up to 50% as mild, reduction up to 70% as moderate and diameter reduction over 70% as severe. As there is no standardized classification for vasospasm severity, we combined measuring systems from previous studies with physicians’ assessment [11, 12].

The percent-fold changes in vessel diameter were calculated as a proportional change in vessel diameter pre- and post-angioplasty, correlated with the original diameter on admission day.

Vessel diameters were measured by a single observer after intervention with comparison to the treating physician’s measurement during the intervention and third-observer measurement upon discrepancy.

### Statistical Analysis

Results are presented as means including standard deviation. A multivariable linear regression model was applied to quantify the percentage of restored diameter pharmacological, mechanical and combined treatments compared to single pharmacological treatment. Age, sex, treatment modality, spastic vessel segment, severity of spasm, and number of previous treatments were included as confounding variables. Variables were chosen by forward selection to optimize coefficient of determination and theoretical reasoning based on clinical experience during EVT procedures. To assess the robustness of the results, a sensitivity analysis was conducted.

The linear regression model was re-estimated while varying the covariates and excluding influential observations (Cook’s distance > 5/n). The effects remained qualitatively unchanged.

A two-sided *p* value of < 0.05 was considered statistically significant. Statistical analysis was performed using SPSS (IBM Corp. (2023). *IBM SPSS Statistics für Windows* (Version 29.0). Figure generation was performed using GraphPad Prism (GraphPad Software, *GraphPad Prism,* Version 10.6.1. San Diego).

Prior to interpreting the results, the assumptions of linear regression were examined. Visual inspection of the Q–Q plot and histogram suggested that the residuals were approximately normally distributed. The scatterplot of standardized residuals versus standardized predicted values indicated no pattern, supporting the assumption of homoscedasticity. The Durbin–Watson statistic was 1.88, indicating no violation of the assumption of independent errors. Multicollinearity diagnostics showed acceptable values for all predictors (VIFs < 5; Tolerance > 0.20). No influential cases were detected, as Cook’s Distance values did not exceed 1.

## Results

Results are presented as means with standard deviation (SD), standard error (SE) for linear regression model or as absolute numbers with percentage of population.

60 patients were included with a total of 228 EVT interventions. The mean time interval between the ictus and the performance of interventional EVT was 6.98 days (SD ± 3.7 days). Patients received a mean of 3.8 interventions. In total, 798 vessel segments were documented of which 614 could be graded for severity and 628 vessel segments were treated and consecutively included in statistical analysis. 320 segments were treated with a single pharmacological agent; 142 segments were treated with combined medication. 44 vessel segments received Stent-Retriever-Angioplasty combined with one pharmacological agent; 19 segments received Stent-Retriever-Angioplasty with dual pharmacological agent administration. 12 vessel segments received Stent-Retriever-Angioplasty with no pharmacological agents. 54 vessel segments received TBA treatment combined with single pharmacological agent, 10 vessel segments received TBA treatment combined with dual pharmacological agents, and 27 vessel segments received TBA treatment without the use of pharmacological agents. 77 spastic vessel segments were not treated.

Mean initial vessel reduction regardless of location showed a mean diameter reduction of 53.52% (SD ± 15.38%) due to vasospasm.

Vasospasm presentation documented the initial presentation suspect of vasospasm with additional imaging (e.g., CCT) not shown but present in all initial vasospasm presentations.

**Fig. 1:**
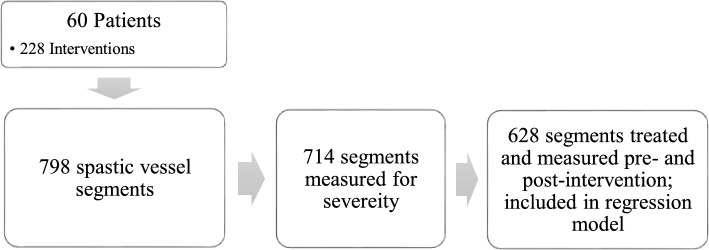
Flow diagram of study group. The diagram shows the number of patients included, the number of interventions and the number of vessel segments extracted for quantitative analysis

Additional data are shown in Table 1 and Fig. 1.

**Table 1:** Vasospasm characteristics

Vasospasm presentation	
Worsening of neurologic condition	18 (7.9%)
CCT	9 (3.9%)
TCD	37 (16.2%)
Angiography after EVT	155 (68%)
Aneurysm Treatment	4 (1.8%)
Other	5 (2.4%)
Total	228
Vasospasm Location—Hemisphere	
Right Hemisphere	99 (50.0%)
Left Hemisphere	350 (43.9%)
Basilar	49 (6.1%)
Total	798
Vasospasm Location – Vessel Segment	
Distal ICA	49 (6.1%)
A1 Segment	215 (26.9%)
A2 Segment	75 (9.4%)
Peripheral ACA	33 (4.1%)
M1 Segment	144 (18.0%)
M2 Segment	113 (14.2%)
Peripheral MCA	31 (3.9%)
P1 Segment	59 (7.4%)
Peripheral PCA	6 (0.8%)
Basilar	49 (6.1%)
V4 Segment	24 (3.0%)
Total	798
Vasospasm Grading	
Mild (< 50% reduction)	259 (36.3%)
Moderate (50–70% reduction)	371 (52.0%)
Severe (> 70% reduction)	84 (11.8%)
Total	714

Vasospasm characteristics Results are presented as total number and percentage. *A1* anterior cerebral artery—A1 segment, *A2* anterior cerebral artery—A2 segment, *ACA* anterior cerebral artery, *ICA* internal carotid artery, *M1* middle cerebral artery—M1 segment, *M2* middle cerebral artery—M2 segment, *MCA* middle cerebral artery, *P1* posterior cerebral artery—P1 segment, *PCA* posterior cerebral artery, *V4* vertebral artery—V4 segment, *CCT* Cranial Computer Tomography, *TCD* transcranial Doppler sonography

The overall model showed a significant increase in diameter restoration with mechanical angioplasty in comparison with Single Med. (constant). TBA increases diameter restoration the most in combination with dual pharmacological therapy. While TBA shows a significant difference in diameter restoration with single or no pharmacological combination, stent-retriever angioplasty only shows a significant difference in diameter restoration with dual pharmacological combination. Linear regression did not demonstrate a significant correlation between the spastic vessel segment and diameter restoration. Linear regression showed no significant correlation between the number of previous segment treatments and diameter restoration. Additional results are displayed in Table 2.

**Table 2:** Multivariate linear regression model

*R*	*R*^2^	Adjusted *R*^2^	Std. Error of the Estimate
.579	.335	.315	.172273

Unstandardized Coefficients		Standardized Coefficients				95% Confidence Interval	
Treatment modality	Regression coefficient B	Std. error	Std. Coefficient Beta	t	p	Lower Bound	Upper Bound
(Constant)	.781	.050	/	15.772	< .001	.684	.878
Sex—Male	.007	.016	.014	.403	.687	−.025	.038
Age	.000	.001	−.011	−.302	.763	−.002	.001
Modality (Comparator: Single Med.)							
Dual Med	.044	.018	.089	2.441	.015	.009	.080
TBA single	.158	.038	.155	4.158	< .001	.083	.233
Single Med. + TBA	.216	.028	.291	7.707	< .001	.161	.270
Dual Med. + TBA	.284	.057	.171	4.971	< .001	.172	.396
Stent single	.098	.052	.064	1.894	.059	−.004	.199
Single Med. + Stent	.055	.029	.067	1.920	.055	−.001	.111
Dual Med. + Stent	.215	.041	.178	5.193	< .001	.134	.297
Vessel Segment (Comparator: Distal ICA)							
A1	.000	.033	−.001	−.008	.993	−.065	.064
A2	.010	.037	.015	.271	.786	−.062	.082
Distal ACA	.004	.049	.004	.089	.929	−.092	.101
M1	−.038	.032	−.071	−1.180	.239	−.101	.025
M2	−.007	.035	−.013	−.210	.833	−.077	.062
Distal MCA	.030	.053	.023	.567	.571	−.075	.135
P1	−.011	.039	−.013	−.274	.784	−.086	.065
Distal PCA	.059	.106	.020	.560	.576	−.149	.267
Basilar	.039	.040	.043	.969	.333	−.040	.119
V4 Segment	.060	.047	.052	1.275	.203	−.033	.154
Spasm Severity (Comparator: Mild Spasm)							
Moderate Spasm	−.184	.015	−.442	−11.933	< .001	−.214	−.154
Severe Spasm	−.247	.024	−.378	−10.208	< .001	−.294	−.199
No. of previous treatments	−.005	.006	−.029	−.821	.412	−.016	.006

Regression coefficients are depicted as percentages of diameter increase compared to single medical treatment (constant). Age, (male) sex, vessel segments, severity of spasm and number of previous treatments as confounding variables. Moderate spasm—50–70% diameter reduction, severe spasm—> 70% diameter reduction. *A1* anterior cerebral artery—A1 segment, *A2* anterior cerebral artery—A2 segment, *ACA* anterior cerebral artery, *ICA* internal carotid artery, *M1* middle cerebral artery—M1 segment, *M2* middle cerebral artery—M2 segment, *MCA* middle cerebral artery, *P1* posterior cerebral artery—P1 segment, *PCA* posterior cerebral artery, *V4* vertebral artery—V4 segment

**Fig. 2:**
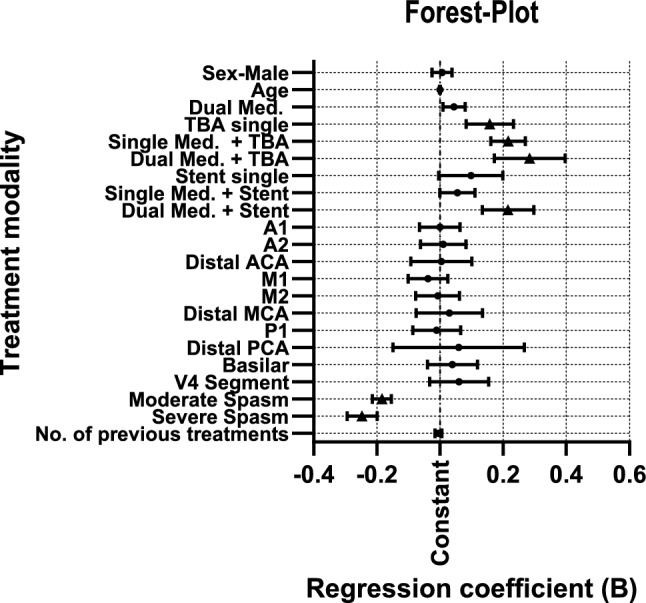
Forest plot of linear regression model. Displayed are the results of the linear regression analysis illustrating the influence of covariates/confounding factors, including 95% confidence intervals, compared to single medical treatment (constant, shown as vertical dotted baseline). Statistically significant results (*p* < 0.05) are highlighted with a triangle. *Moderate spasm* – 50–70% diameter reduction, *severe spasm*—> 70% diameter reduction. *TBA* transluminal balloon angioplasty; *A1/A2* segments of the ACA (anterior cerebral artery); *M1/M2* segments of the *MCA* (middle cerebral artery); *P1* = segment of the PCA (posterior cerebral artery); *V4* = segment V4 of the vertebral artery

Furthermore, subgroup analysis of pharmacological treatment showed no significant effect with different dosages on therapeutic efficacy (not displayed in table). Regression is also shown in Fig. 2.

A total number of 19 (2.4%) complications were documented during 628 interventional procedures including intracranial adverse outcomes associated with endovascular therapy and hemodynamic events as listed below. In 4 (0.5%) cases angioplasty was not successful with the consecutive decision to implant a definitive stent. In 1 (0.1%) case a paradox stent reaction with M2 spasm was documented. Spasm was dissolved by repeated stent-retriever angioplasty. In 2 (0.3%) cases vessel occlusion after micro-catheter manipulation was documented after which the vessels received successful EVT. In 2 (0.3%) cases vessels reacted with paradox spasm after pharmaceutical application. In 4 (0.5%) cases a thromboembolic vessel occlusion was recorded, 3 vessels were treated by mechanical thrombectomy, and 1 vessel received heparin therapy. All thromboembolic occlusions were dissolved successfully. In 3 (0.4%) cases intracranial pressure spikes were documented on monitor after application of pharmaceutical agent. In 3 (0.4%) cases pharmaceutical EVT failed.

## Discussion

Our study presents new data which highlights limited differences between therapeutic combinations in endovascular EVT. Our data suggest better angiographic outcomes with mechanical angioplasty, especially TBA with the option of combining pharmacological and mechanical treatment modalities. The combination of TBA and pharmacological application has been suggested by previous studies [6]; nevertheless, the use of TBA has been limited to larger vessels due to a possible rise in complications [10]. This study adds a potential benefit of comparing TBA with single or dual pharmaceutical therapy which has been proposed by other authors with single pharmaceutical therapy combination [13, 14]. Furthermore, the use of stent-retriever angioplasty seems to be most effective with a dual pharmacological combination but the quantitative data of this treatment group are limited. Kwon et al. also reported better angiographic outcomes with the use of a single vasodilator [15]. The use with dual pharmacological application has not been investigated so far but it is possible that nimodipine and papaverine work synergistically by targeting different cellular structures [15]. In our center, past experiences show better treatment efficacy. This improvement could be due to the fact that vasodilators can be injected during the deployment of the stent, allowing the drug to reach the distal vasculature. The improvement could also be biased by a higher dose of medication being administered. Although calcium antagonists like verapamil are also utilized in intra-arterial spasm treatment, our center mainly uses nimodipine and papaverine. Although papaverine has had mixed results in studies we experienced better peripheral spasm relief whereas nimodipine showed good angiographic outcome on central lesions. One complication was recorded after stent implantation. This is in accordance with previous studies investigating the feasibility and safety of stent-retriever angioplasty [7, 16]. It is important to recognize that stent-retriever angioplasty seems less effective than TBA based on vasodilatory effect alone but offers different advantages due to the smaller radial force exerted. Stent-Retriever-Angioplasty may provide an additional avenue for accessing smaller vessels, such as the A2, M2, and P1 territories, thereby optimizing flow restoration in these regions [7]. With the combination of mechanical and pharmacological angioplasty the single use of agents may be of short effect, although Zaeske et al. investigated the underestimation of the vasodilatory effect in immediate angiographic control [17]. Studies investigating the lasting effect of mechanical angioplasty are warranted.

As vasospasms are still a predictor of higher mortality in patients [18], methods to enhance treatment and studies to evaluate effective treatment methods are needed. Ideally, individual patient treatment with a range of endovascular EVT techniques could improve overall patient outcome. Our study adds a large group of vessel-segment treatments with comparison between pharmaceutical and mechanical EVT. Other studies have investigated the efficacy and safety of stent-retriever angioplasty-assisted EVT but current literature still lacks data on the efficacy of stents when compared and combined with pharmaceutical treatment [7, 8, 16].

In a survey published by Guenego et al., a discrepancy between the estimated effectiveness of mechanical angioplasty and the routine of its clinical use was shown: Physicians estimated, in their experience, mechanical angioplasty to be more effective in vasospasm treatment than pharmaceutical treatment alone, but the use of mechanical angioplasty to treat spastic vessels was usually reserved as rescue therapy after pharmaceutical management [3]. As further mentioned in our limitations, it remains unclear whether the observed angiographic improvements translate into clinically relevant outcomes. Vasospasm and DCI remain clinically challenging with different treatment options having yielded only small results but as endovascular EVT remains relevant in clinical practice possible improvements ought to be investigated.

The retrospective study design allowed us to include a large population of treated vessel segments at the expense of limiting our extracted data and the quality of statistical analysis. No standardized treatment regime was followed as choice of treatment was up to the treating neuroradiologist. As there are no clear treatment regimens and guidelines to endovascular vasospasm treatment, this adds to the significant heterogeneity between treatment modalities. [1, 3] Different dosages of medication were used and although subgroup analysis showed no significant effect with different dosages, this adds to a high treatment bias, also limiting the external validity of our results. The definition of vasospasm and the rationale for interventional treatment also differs among authors [11, 12], especially when grading the severity of spasm which leads to incongruent treatment indications and more inaccurate analysis [18, 19] No standardized intra- or interobserver variability analysis was performed, which raises the possibility of measurement errors.

We used the severity of spasm as a confounder to limit the bias of larger arterial segments potentially having less vasospasm, thus overestimating the therapeutic effect of angioplasty. It would have been possible to choose the reduction of spasm as our primary endpoint, but we chose the restoration of the original diameter to accurately assess the effect on arterial diameter. Good post-interventional contrast enhancement in the territory peripheral to the treated segment was used as a surrogate for effective EVT.

It is important to note that our study did not compare treatment methods to patient outcome, post-interventional infarction rate or CT perfusion imaging and no follow-up data was obtained. Therefore, our study does not allow a conclusion on whether therapeutic modality has an influence on a clinically meaningful outcome, limiting our relevance. This was mainly because outcome after aneurysmal subarachnoid hemorrhage is influenced by many contributing factors such as ICU management, epidemiological predictors and an overall pathophysiology that has not been fully understood yet. However, the treatment of vasospasm in our clinic is subject to strict angiographic monitoring until the condition has been resolved. As a result, we have elected to use arterial diameters as a surrogate parameter for the outcome following interventional EVT and DCI.

## Conclusion

TBA remains most effective for large-vessel vasospasm. Stent-retriever angioplasty remains less effective than TBA but may present more effective than pharmacological treatment alone balancing the technical challenges and risk profile. Sole pharmaceutical treatment may still be first-line treatment but prove less effective for refractory vasospasm. Trials with structured treatment regimens are warranted to evaluate clinical outcome and validate these findings in prospective setting.

## Supplementary Information

Below is the link to the electronic supplementary material.

Supplementary file1 (DOCX 17 KB)
