# Supplementary material for: Pharmaceutically Assisted Angioplasty to Relieve Cerebral Vasospasm after Aneurysmal Subarachnoid Hemorrhage—A Retrospective, Single-Center, Observational Comparison of Established and New Treatment Techniques
Source: Cardiovasc Intervent Radiol. 2026 Feb 16;49(4):800–8. doi: 10.1007/s00270-026-04367-w (PMC13021696; doi:10.1007/s00270-026-04367-w)
Supplement: Supplementary file 1 — Supplementary file1 (DOCX 17 KB) [file 270_2026_4367_MOESM1_ESM.docx]

**Supplemental**

**Patient data**

Results are presented as means with standard deviation (SD) or as absolute numbers with percentage of population.

Additional data is shown in table (1).

**Supplemental Table 1. Clinical information of patient population**

| Age (years) | 50.63 ± 11.32 |
| --- | --- |
| **Sex** |  |
| Female | 46 (76.7%) |
| Male | 13 (23.3%) |
| **Number of aneurysms per patient** |  |
| 1 | 41 (68.3%) |
| 2 | 7 (11.7%) |
| 3 | 2 (3.3%) |
| 4 | 4 (6.7%) |
| 5 | 1 (1.7%) |
| 6 | 1 (1.7%) |
| No diagnosed aneurysm | 4 (6.7%) |
| **Site of aneurysm** |  |
| Media bifurcation | 22 (25.6%) |
| ACOM | 16 (18.6%) |
| ICA | 12 (14.0%) |
| Pericallosal artery | 7 (8.1%) |
| Basilar | 2 (2.3%) |
| PCOM | 5 (5.8%) |
| MCA – Not Media bifurcation | 4 (4.7%) |
| ACA | 3 (3.5%) |
| Vertebral artery | 3 (3.5%) |
| Other | 8 (9.3%) |
| **Hunt&Hess-grade at admission** |  |
| 1 | 4 (6.7%) |
| 2 | 12 (20.0%) |
| 3 | 11 (18.3%) |
| 4 | 11 (18.3%) |
| 5 | 17 (28.3%) |
| Not documented | 5 (8.3%) |
| **SAH treatment method** |  |
| Conservative | 7 (11.7%) |
| EVD implantation | 47 (78.3%) |
| Operative evacuation | 2 (3.3%) |
| EVD + operative evacuation | 4 (6.7%) |
| **Aneurysm treatment method** |  |
| Clipping | 3 (3.7%) |
| Endovascular at physician`s discretion | 68 (82.9%) |
| Incidental, non-culprit aneurysm not treated | 11 (13.4%) |
| **Anti-platelet-agents** |  |
| Aspirin | 8 (13.3%) |
| Aspirin + Ticagrelor | 13 (21.7%) |
| Others | 3 (5.1%) |
| No anti-platelet medication | 36 (60.0%) |
| **Time in days until onset of vasospasm** | 6.98 ± 3.92 |
| **Number of angiographies performed for vasospasmolysis during stay** | 3.8 ± 2.46 |

**Supplemental Table 1.** Clinical information of the selected patients. Results are presented as means + standard deviation (SD) or total number and percentage. *ACOM* anterior communicating artery, *ICA* internal carotid artery, *PCOM* posterior communicating artery, *MCA* middle cerebral artery, *ACA* anterior cerebral artery, ASS aspirin, SAH subarachnoid hemorrhage, EVD extra-ventricular drainage
